# Supplementary material for: End Sequence Analysis Toolkit (ESAT) expands the extractable information from single-cell RNA-seq data
Source: Genome Res. 2016 Oct;26(10):1397–410. doi: 10.1101/gr.207902.116 (PMC5052061; doi:10.1101/gr.207902.116)
Supplement: Supplemental Material [file supp_gr.207902.116_Supp_Materials.tar.gz › ESAT-esat_v0.1/doc/html/namespacebroad_1_1core_1_1sequence.html]

ESAT: Package broad.core.sequence


ESAT


- Main Page
- Related Pages
- Packages
- Classes
- Files

- Packages

All Classes Namespaces Files Functions Variables Enumerator Pages

- broad
- core
- sequence

Classes

Package broad.core.sequence

|  |  |
| --- | --- |
| Classes | |
| class | AbstractFastaParser |
|  | |
| class | Extractor |
|  | |
| interface | FastaHandler |
|  | |
| class | FastaParser |
|  | |
| class | FastaQualityBinaryIO |
|  | |
| interface | FastaQualityHandler |
|  | |
| class | FastaQualityIndex |
|  | |
| class | FastaQualityParser |
|  | |
| class | FastaSequenceIO |
|  | |
| class | Sequence |
|  | |
| class | SequenceRegion |
|  | |
| class | SequenceRegionWindow |
|  | |
| class | SequenceUtils |
|  | |
| class | TranscribedRegions |
|  | |
| class | TranscribedSequenceComposition |
|  | |
| class | WindowSlider |
|  | |


---

Generated on Fri Aug 15 2014 15:10:55 for ESAT by  

 1.8.7
